# Astaxanthin Suppresses PM2.5-Induced Neuroinflammation by Regulating Akt Phosphorylation in BV-2 Microglial Cells

**DOI:** 10.3390/ijms21197227

**Published:** 2020-09-30

**Authors:** Ryeong-Eun Kim, Chan Young Shin, Seol-Heui Han, Kyoung Ja Kwon

**Affiliations:** 1 Department of Neuroscience, School of Medicine, Konkuk University, Seoul 05029, Korea; ryoung_2@naver.com (R.-E.K.); alzdoc@naver.com (S.-H.H.); 2 Department of Pharmacology, School of Medicine, Konkuk University, Seoul 05029, Korea; chanyshin@kku.ac.kr; 3 Department of Neurology, Konkuk Hospital Medical Center, 120-1 Neungdong-ro, Gwangjin-Gu, Seoul 05030, Korea

**Keywords:** PM2.5, microglia, inflammation, polarization, astaxanthin

## Abstract

Air pollution has become one of the most serious issues for human health and has been shown to be particularly concerning for neural and cognitive health. Recent studies suggest that fine particulate matter of less than 2.5 (PM2.5), common in air pollution, can reach the brain, potentially resulting in the development and acceleration of various neurological disorders including Alzheimer’s disease, Parkinson’s disease, and other forms of dementia, but the underlying pathological mechanisms are not clear. Astaxanthin is a red-colored phytonutrient carotenoid that has been known for anti-inflammatory and neuroprotective effects. In this study, we demonstrated that exposure to PM2.5 increases the neuroinflammation, the expression of proinflammatory M1, and disease-associated microglia (DAM) signature markers in microglial cells, and that treatment with astaxanthin can prevent the neurotoxic effects of this exposure through anti-inflammatory properties. Diesel particulate matter (Sigma-Aldrich) was used as a fine particulate matter 2.5 in the present study. Cultured rat glial cells and BV-2 microglial cells were treated with various concentrations of PM2.5, and then the expression of various inflammatory mediators and signaling pathways were measured using qRT-PCR and Western blot. Astaxanthin was then added and assayed as above to evaluate its effects on microglial changes, inflammation, and toxicity induced by PM2.5. PM2.5 increased the production of nitric oxide and reactive oxygen species and upregulated the transcription of various proinflammatory markers including Interleukin-1β (IL-1β), Interleukin-6 (IL-6), Tumor necrosis factor α (TNFα), inducible nitric oxide synthase (iNOS), triggering receptor expressed on myeloid cells 2 (TREM2), Toll-like receptor 2/4 (TLR2/4), and cyclooxygenase-2 (COX-2) in BV-2 microglial cells. However, the mRNA expression of IL-10 and arginase-1 decreased following PM2.5 treatment. PM2.5 treatment increased c-Jun N-terminal kinases (JNK) phosphorylation and decreased Akt phosphorylation. Astaxanthin attenuated these PM2.5-induced responses, reducing transcription of the proinflammatory markers iNOS and heme oxygenase-1 (HO-1), which prevented neuronal cell death. Our results indicate that PM2.5 exposure reformulates microglia via proinflammatory M1 and DAM phenotype, leading to neurotoxicity, and the fact that astaxanthin treatment can prevent neurotoxicity by inhibiting transition to the proinflammatory M1 and DAM phenotypes. These results demonstrate that PM2.5 exposure can induce brain damage through the change of proinflammatory M1 and DAM signatures in the microglial cells, as well as the fact that astaxanthin can have a potential beneficial effect on PM2.5 exposure of the brain.

## 1. Introduction

The term “air pollution” refers to a complex mixture of substances, including particulate matter (PM), carbon monoxide, lead, nitrogen dioxide, and sulfur dioxide, among others, known to be detrimental to human health [1]. On the basis of size and aerodynamic properties, PM can be classified as PM10 (≤10 µM), PM2.5 (≤2.5 µM), and ultrafine particulate matter (≤0.1 µM) [2]. PM2.5 particles originate from various sources including oil refineries, metal processing facilities, tailpipe and brake emissions from mobile sources, power plants, and residential fuel combustion. PM2.5 is one of the most harmful materials to our health, and for this reason, it is also the most commonly evaluated type of air pollution. Once inhaled, PM2.5 can induce damage throughout the body and is not easily and shortly removed from the body. Most studies on PM2.5 have focused on respiratory and cardiovascular damage, but it is increasingly likely that PM2.5 may affect tissues beyond the respiratory system. PM2.5 enters the human body through various pathways and has been linked to pathological effects in the central nervous system (CNS). PM2.5 can destroy the integrity of the blood–brain barrier (BBB), allowing for increased access to the sensitive neural tissues and increased peripheral systemic inflammation. PM2.5 has also been linked to damage in the olfactory neurons [3] with supporting epidemiological studies of long-term exposure to PM2.5. These particles infiltrate through the lungs, resulting in neurodegenerative diseases, type-2 diabetes, obesity, respiratory infection, cardiovascular disease, systemic inflammation, and metabolic syndrome [1,4]. Recently, a study reported that PM2.5 can gain access to the gastrointestinal tract via the gut microbiota, exacerbating injuries to the CNS and inducing various neurodegenerative diseases including Alzheimer’s disease (AD) [5].

Microglia are the resident innate immune cells of the central nervous system that play a critical role in both normal and pathological conditions. Neuroinflammation following microglia activation is considered a double-edged sword that can exert either neuroprotective or neurotoxic effects in the brain [4,5]. Microglia are the first line of host defense against various neuronal injuries and/or other stimuli, releasing either proinflammatory mediators such as proinflammatory cytokines, reactive oxygen species, nitric oxide (NO), nerve growth factor, and chemotactic cytokines inducing the neuronal toxicity or anti-inflammatory factors preventing neuronal damage [6]. Microglia exert beneficial effects in mediating the neuronal development and brain homeostasis maintenance. When the brain is injured or affected by brain diseases, “resting” microglia transform into “activated microglia” through a series of cellular and molecular changes [7,8]. Different stimuli produce different microglial phenotypes that can be classified as classical, either M1 or M2. M1 microglia increase proinflammatory responses in the brain while M2 microglia induce neuroprotective effects, playing an anti-inflammatory role. Previously, many studies have demonstrated that an imbalance in microglial polarization through the excessive activation of M1 microglia or inhibition of M2 microglia may promote the development and progression of various neuronal disorders and neurodegenerative diseases such as AD. Recent studies suggest the existence of disease-associated microglia (DAM), a recently identified subgroup of CNS microglia found in neurodegeneration, chronic neuroinflammatory states, and advanced aging [6,9]. This is a distinct phenotype that challenges the classical view of the microglia polarization, proinflammatory M1, and immunosuppressive M2 state. In disease conditions including AD, homeostatic microglial phenotype changes into proinflammatory DAM and anti-inflammatory DAM through triggering receptor expressed on myeloid cells 2 (TREM2) checkpoint. DAM has a dual role, immunosuppressive and inflammatory, in disease progression, including AD. These roles involve the TREM2 expression and control microglial activity and survival [6], while several variants of TREM2 increase the developing late-onset AD. In the earlier stage of AD, increased levels of TREM2 may be a neuroprotective role associated with amyloid clearance. In more advanced stages of the disease, involving TREM2-expressing microglia, DAM has been shown to cause extensive inflammation and neurodegeneration [10,11].

Therefore, microglia-mediated neuroinflammation is a hallmark of various neurodegenerative diseases, including Alzheimer’s disease (AD), Parkinson’s disease (PD), and amyotrophic lateral sclerosis (ALS) [12,13,14]. Modulation of microglial polarization toward the anti-inflammatory DAM phenotype could be an effective therapeutic and preventive strategy for the treatment of neuroinflammatory and neurodegenerative diseases resulting from various environmental factors, including air pollution, especially PM2.5. However, it is still unclear as to whether exposure to PM2.5 directly results in the polarization into proinflammatory M1 and DAM phenotype.

Astaxanthin (3,3′-dihydroxy-β,β′-carotene-4,4′-dione, ATX) is a natural xanthophyll carotenoid found in many marine organisms including *Haematococcus pluvialis, Chlorella zofingiensis, Chlorococcum*, and *Phaffia rhodozyma* [15,16]. It has been reported to exert anti-inflammatory, antioxidant, and neuroprotective effects [17,18,19], and the results from various experimental models have shown that these effects are associated with the reduced expression of pro-inflammatory cytokines and the reduced production of reactive oxygen species (ROS) and free radicals [17,20]. Owing to its multiple beneficial effects, ATX is under investigation for its protective effects against inflammatory and immunomodulatory responses, diabetes, cardiovascular damage, neuronal damage, aging, and cancer [15,20,21]. In general, ATX exerts its anti-inflammatory function by reducing the expression of pro-inflammatory cytokines through the inhibition of Nuclear factor-κB (NF-κB) activation [22,23], as well as its antioxidative effects via a NF-E2-related factor 2 (Nrf2)-mediated mechanism [17,24]. However, although ATX plays a critical role in the prevention of oxidative stress and inflammatory response, the effects of ATX on particulate matter-induced -microglial activation are yet to be determined. Therefore, we sought to demonstrate the mechanism underlying PM2.5-induced brain damage and evaluate the effect of ATX treatment on the microglial activation. Our results suggest the novel therapeutic strategy for preventing particulate matter-induced brain damage.

## 2. Results

### 2.1. PM2.5 Increased Neuroinflammation via Microglial Activation

**Figure 1:**
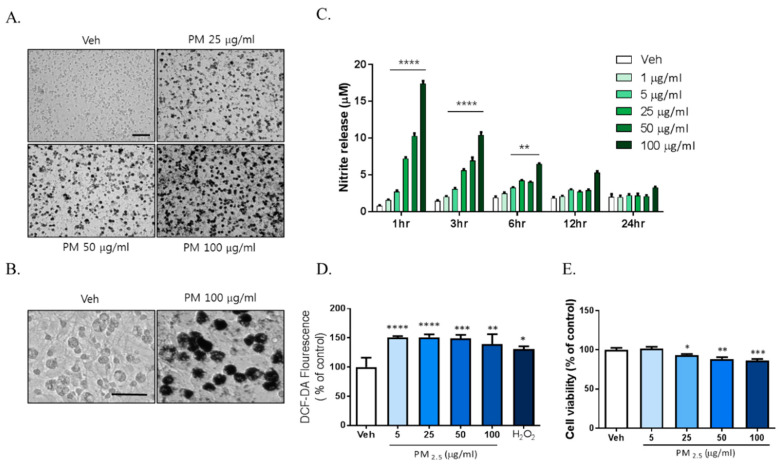
The toxic effects of PM2.5 treatment on cultured glial cells. Rat primary cultured glial cells were treated in Dulbecco’s modified Eagle’s medium (DMEM)/F12 with increasing concentration of PM2.5 (5, 25, 50, 100 µg/mL) for 24 h. (**A**) Toxic effects of PM2.5 in cultured glial cells. After 24 h, cell morphology was measured by phase contrast imaging. Bar size = 50 μm. (**B**) High magnification. Scale bar = 50 μm. (**C**) Nitrite production in the medium was assessed using Griess reagent. Rat primary cultured glial cells were incubated in DMEM/F12 with increasing concentration of PM2.5 (1, 5, 25, 50, 100 µg/mL) for each incubation time. (**D**) Intracellular reactive oxygen species (ROS) production was measured by 2′,7′-Dichlorodihydrofluorescein diacetate (H_2_DCF-DA) fluorescence (490/530 nm). (**E**) Effect on cell viability was measured by 3-[4,5-dimethylthiazol-2-yl]-2,5-diphenyl-tetrazolium bromide (MTT) assay. Rat primary cultured glial cells were incubated in DMEM/F12 with increasing concentration of PM2.5 (5, 25, 50, 100 µg/mL) for 24 h. Data represent means ± SEM. (*n* = 4) * *p* < 0.05, ** *p* < 0.01, *** *p* < 0.001, **** *p* < 0.0001; veh (0.1% DMSO) vs. PM2.5.

Previously reported data showed that exposure to PM2.5 increases inflammation and oxidative stress in respiratory systems including in the lung and alveoli. We investigated whether PM2.5 exposure increased inflammation in brain cells such as the microglia. To investigate the effects of PM2.5 on brain cells, we used diesel particulate matter (DPM) provided by Sigma-Aldrich Ltd. as a PM2.5 alternative. Mixed rat glial cells were treated with 5, 25, 50, and 100 µg/mL DPM dissolved in 0.02% DMSO and sonicated for 30 min freshly. We observed significant accumulation of the particulate matter around the microglia with density closely related to dose from 25 µg/mL PM2.5 to 100 µg/mL PM2.5 (Figure 1A). To investigate the effects of PM2.5 on microglial activation, we evaluated the effects of PM2.5 treatment on the inflammatory response in BV-2 microglial cells. PM2.5 treatment increased ROS and NO production in BV-2 microglial cells. Interestingly, PM2.5 treatment also significantly upregulated NO release from 1 h (Figure 1B). However, lipopolysaccharide (LPS; O111:B4), a well-known as NO and inflammatory response inducer, did not exhibit the same results at that time (data not shown). PM2.5 treatment increased ROS production, which was measured using 2′,7′-Dichlorofluorescin Diacetate (DCF-DA) fluorescence (Figure 1C). To evaluate the effect of PM2.5 treatment on cell viability, we treated mixed rat glial cells with different concentrations of PM2.5 (5, 25, 50, 100 µg/mL) and measured the cell viability by 3-[4,5-dimethylthiazol-2-yl]-2,5-diphenyl-tetrazolium bromide (MTT) assay. PM2.5 treatment reduced cell viability in a dose-dependent manner (Figure 1D). These results indicate that PM2.5 exposure results in the rapid induction of the inflammatory response and microglial activation.

### 2.2. PM2.5 Increased the Transcription of Proinflammatory M1 and DAM Phenotype Molecules, and Decreased the Expression of Anti-Inflammatory Genes

**Figure 2:**
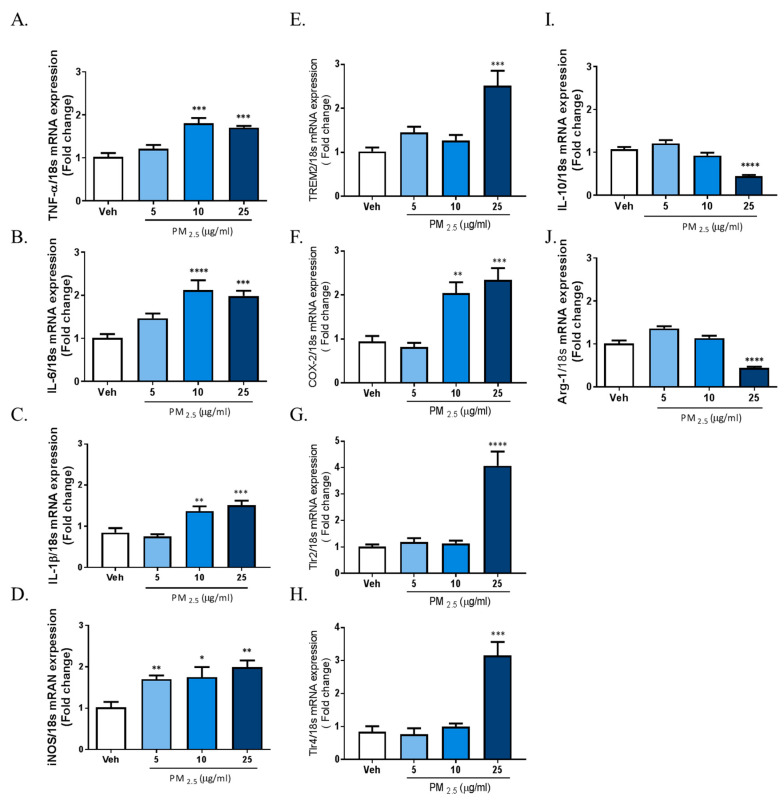
Effect of PM2.5 treatment on the mRNA expression of proinflammatory M1 and disease-associated microglia (DAM) phenotype molecules and anti-inflammatory markers in BV-2 microglial cells. BV-2 microglial cells were incubated in DMEM/F12 increasing concentration of PM2.5 (5, 10, 25 µg/mL) for 1 h and 24 h. (**A**–**D**) Effect on the mRNA expression of proinflammatory M1 marker genes. (**E**–**H**) Effect of the mRNA expression of DAM phenotype. (**I**,**J**) Effect on the mRNA expression of anti-inflammatory markers. Expression of total cellular of TNFα, IL-6, IL-1β, IL-10, arginase-1, triggering receptor expressed on myeloid cells 2 (TREM2), COX-2, TLR2, and TLR4 mRNA quantified by qRT-PCR. Glyceraldehyde-3-phosphate dehydrogenase (GAPDH) levels were measured as an internal control. Data represent means ± SEM. (*n* = 4) * *p* < 0.05, ** *p* < 0.01, *** *p* < 0.001, **** *p* < 0.0001; veh vs. PM2.5.

The results above demonstrated that PM2.5 treatment induced inflammatory responses in rat glial cells. Therefore, we investigated whether microglia phenotype change can be induced by exposure to PM2.5 in BV-2 microglial cells. We measured the gene expression profiles of various inflammatory cytokines as M1 markers; the gene expression of Interleukin-10 (IL-10) and arginase-1 as anti-inflammatory marker genes; and the gene expression of TREM2, TLRs, and COX-2 as proinflammatory DAM phenotype markers using qRT-PCR. To measure the mRNA expression of total cellular TNFα, IL-6, IL-1β, iNOS, and COX-2, we incubated BV-2 microglial cells in Dulbecco’s modified Eagle’s medium (DMEM)/F12 with increasing concentrations of PM2.5 (5, 10, 25 µg/mL) for 4 h. To measure the gene expression of IL-10, arginase-1, TREM2, TLR2/4, and COX-2, we treated cells with PM2.5 (5, 10, 25 µg/mL) for 24 h. Exposure to PM2.5 increased the gene expression of inflammatory M1 markers such as IL-1β, TNFα, IL-6, and iNOS, as well as the gene expressions of proinflammatory DAM phenotype factors such as TREM2, TLR2/4, and COX-2, while reducing the expression of anti-inflammatory genes including IL-10 and arginase-1 (Figure 2). These results indicated that PM2.5-inudced inflammation is associated with the change of microglia signature via the upregulation of the proinflammatory molecules. Taken together, our data suggest that PM2.5 promotes the excessive inflammation through signature change into the proinflammatory M1 and DAM phenotype in microglial cells.

### 2.3. ATX Treatment Inhibited PM2.5-Induced Neuroinflammation in BV-2 Microglial Cells

**Figure 3:**
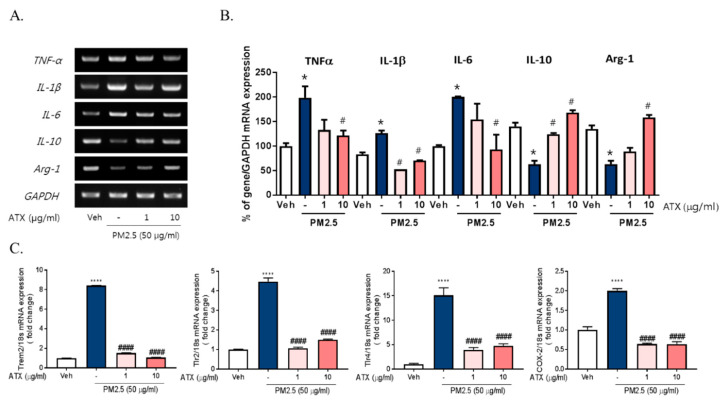
Effects of astaxanthin on PM2.5-induced microglial signature in BV-2 microglial cells. BV-2 microglial cells were treated with ATX (1, 10 µg/mL, astaxanthin). After 4 h, PM2.5 (50 µg/mL) was added in cultured media for 4 h and 24 h. (**A**) Expression of total cellular of TNFα, IL-6, IL-1β, IL-10, and arginase-1 mRNA were measured by RT-PCR. GAPDH levels were measured as a loading control. (**B**) The graph represents quantification data of RT-PCR. (**C**) Gene expression of DAM phenotype was measured by qRT-PCR. Data represent means ± SEM. (*n* = 4) * *p* < 0.05, **** *p* < 0.0001, veh vs. PM2.5, # *p* < 0.05, #### *p* < 0.0001; PM2.5 vs. PM2.5 + ATX.

There are accumulating pieces of evidence supporting the application of ATX as potential antioxidant and anti-inflammatory compounds [15,17,20]. Here, we examined the protective effects of ATX on PM2.5-induced neuroinflammation in BV-2 microglial cells. To investigate the protective effects of astaxanthin on PM2.5-induced neuroinflammation, we added ATX to culture media of BV-2 microglial cells for 4 h and then these cells were treated with PM2.5. ATX treatment decreased the expression of various inflammatory cytokines including IL-1β, TNFα, and IL-6 increased by PM2.5 treatment as the level of vehicle control (Figure 3). Additionally, the gene expression of TREM2, TLR2/4, and COX-2 increased by PM2.5 treatment was inhibited by ATX treatment, whereas the gene expression levels of IL-10 and arginase-1 decreased by PM2.5 were restored by ATX treatment. These results demonstrate that ATX can inhibit PM2.5-induced neuroinflammation via reformulating anti-inflammatory microglia signature.

**Figure 4:**
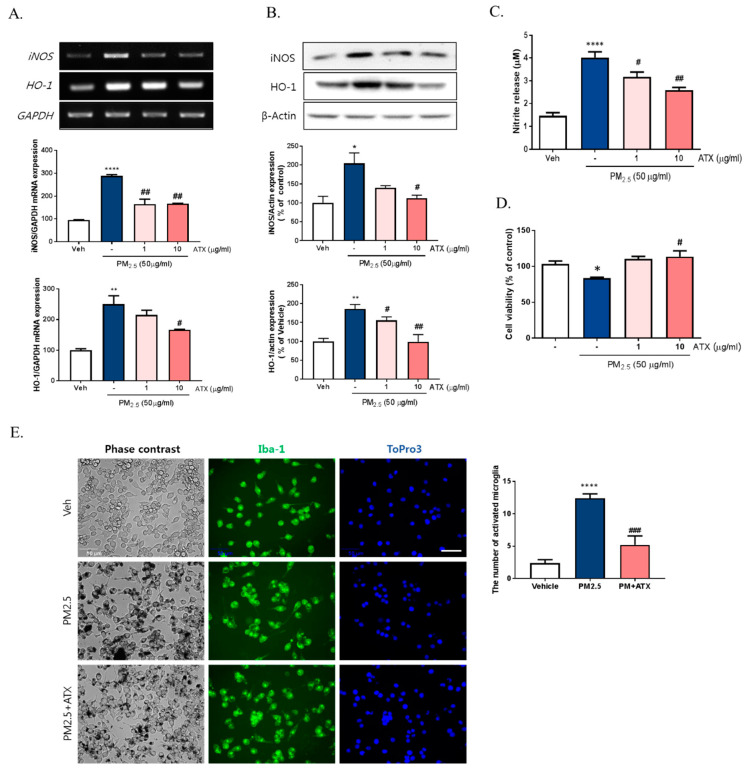
Effects of astaxanthin on PM2.5-induced microglial inflammatory response in BV-2 microglial cells. BV-2 microglial cells were treated with ATX (1, 10 µg/mL, astaxanthin). After 4 h, PM2.5 (50 µg/mL) was treated in cultured media for 24 h. (**A**) Expression of total cellular of iNOS and heme oxygenase-1 (HO-1) mRNA were measured by RT-PCR. GAPDH levels were measured as a loading control. (**B**) The level of total cellular iNOS and HO-1 protein was determined by Western blot using specific antibody against iNOS and HO-1. β-Actin levels were measured as a loading control. (**C**) Nitrite production was measured using Griess reagent. (**D**) Cell viability was measured by MTT assay. (**E**) Microglial activation by immunocytochemistry using the microglial-specific antibody Iba-1. Bar size = 50 μm. Data represent means ± SEM. (*n* = 4) * *p* < 0.05, ** *p* < 0.01, **** *p* < 0.0001; veh vs. PM2.5, # *p* < 0.05, ## *p* < 0.01, ### *p* < 0.001, PM2.5 vs. PM2.5 + ATX.

To further verify the inhibitory effects of ATX on PM2.5-induced neuroinflammation in BV-2 microglial cells, we examined cell viability, NO production, iNOS, and heme oxygenase-1 (HO-1) mRNA and protein expression. PM2.5 exposure in BV-2 microglial cells increased NO production, which was inhibited following ATX treatment. Increasing NO production following PM2.5 exposure was supported by increases in the expression of iNOS at both the transcript and protein levels, with ATX inhibiting both this iNOS and the inflammatory cytokine expression associated with increased neuroinflammation. In addition, for HO-1, a stress-induced protein, transcription and protein expression was significantly increased following PM2.5 treatment, and ATX treatment was shown to inhibit both the transcription and expression of the HO-1 protein, reversing another effect of PM2.5 treatment (Figure 4A–C). PM2.5 decreased cell viability, while ATX treatment prevented PM2.5-induced cell toxicity in BV-2 microglial cells. In addition, we examined whether PM2.5 induces microglial activation using ionized calcium-binding adapter molecule 1 (Iba-1, a microglia-specific marker) staining. These results showed that PM2.5 treatment increased Iba-1 staining in cells, as well as morphological change, confirming our belief that PM2.5 exposure increases microglia activation. However, ATX treatment prevented the increase in the length of the cytoplasmic process induced by PM2.5 treatment in BV-2 microglial cells (Figure 4D,E). Taken together, these results suggest that PM2.5 exposure enhances oxidative stress and inflammation in microglia cells, and ATX treatment can inhibit this PM2.5-induced neuroinflammation.

### 2.4. ATX Decreased PM2.5-Induced pJNK Activation and Increased Akt Phosphorylation

**Figure 5:**
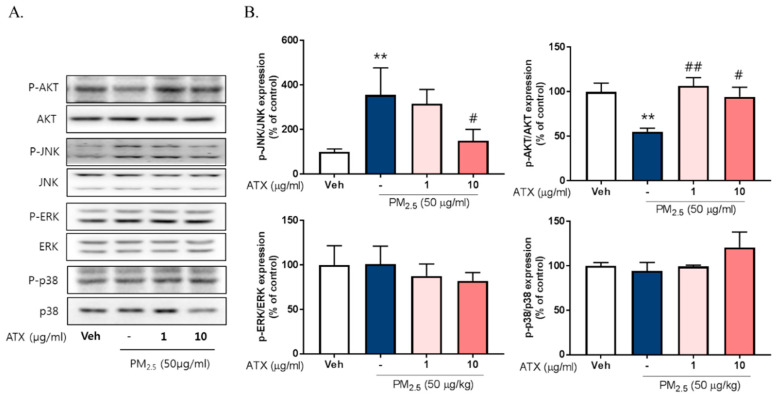
Effects of astaxanthin on PM2.5-induced JNK and Akt phosphorylation in BV-2 microglial cells. (**A**,**B**) Cultured cells were treated with ATX (1, 10 µg/mL, astaxanthin). After 24 h, PM2.5 (50 µg/mL) was treated in cultured media for 30 min and was harvested for Western blot analysis. Immunoblots of lysates were probed with phospho-ERK, phospho-JNK, phospho-p38 antibodies, and phospho-Akt. As a loading control, total ERK/JNK/p38/Akt levels were measured. Data represent means ± SEM. (*n* = 4), ** *p* < 0.01; veh vs. PM2.5, # *p* < 0.05, ## *p* < 0.01; PM2.5 vs. PM2.5 + ATX.

To investigate the signaling pathways associated with PM2.5-induced neuroinflammation, we examined the expression and phosphorylation of various Mitogen-activated protein kinase (MAPK), including Extracellular Signal Regulated Kinase (ERK), p38, and c-Jun NH2-terminal kinase (JNK), using Western blot. PM2.5 exposure increased JNK phosphorylation about threefold but there was no change in p38 or ERK activation. Additionally, PM2.5 exposure decreased Akt phosphorylation while ATX treatment inhibited JNK activation and increased Akt activation (Figure 5). To verify the association between JNK activation and PM2.5-induced inflammatory mediators, we evaluated the effect of SP600125, a JNK inhibitor, on iNOS and HO-1 expression. These results showed that SP600125 treatment (10 µM) inhibited the expression of both proteins in PM2.5-treated cells.

**Figure 6:**
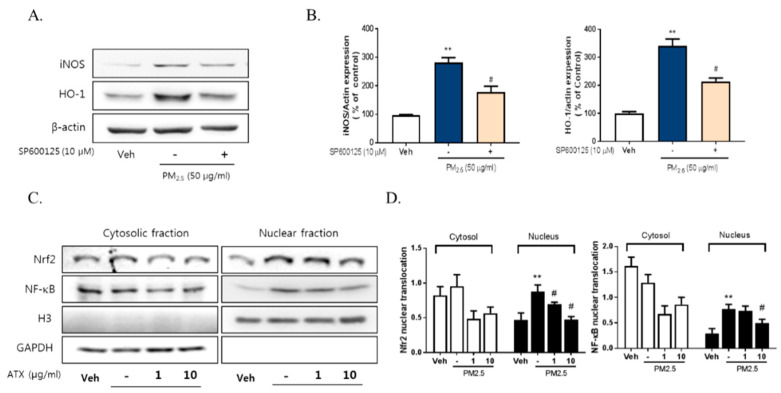
Effects of JNK inhibitor on PM2.5-induced inflammatory mediators in BV-2 microglial cells. BV-2 microglial cells were treated with SP60015 (a specific inhibitor of JNK, 10 µM). After 30 min, PM2.5 (50 µg/mL) was treated in cultured media for 24 h and was harvested for Western blot analysis. (**A**,**B**) Expression of total cellular of iNOS and HO-1 protein. β-Actin levels were measured as a loading control. (**C**) Effects of astaxanthin on PM2.5-induced inflammatory transcription factors in BV-2 microglial cells. BV-2 microglial cells were treated with ATX (1, 10 µg/mL, astaxanthin). After 24 h, PM2.5 (50 µg/mL) was treated in cultured media for 4 h and was harvested for subcellular fraction of NF-E2-related factor 2 (Nrf2) and NF-κB. The nuclear translocation level of Nrf2 and p65 protein was determined by Western blot using specific antibody against Nrf2 and p65. GAPDH for cytosolic fraction and H3 for nuclear fraction levels were measured as a loading control. (**D**) The graph represents quantification data of Western blot. Data represent means ± SEM. (*n* = 4), ** *p* < 0.01; veh vs. PM2.5, # *p* < 0.05; PM2.5 vs. PM2.5 + ATX.

To evaluate the relationship with NF-κB, Nrf2, and neuroinflammation following PM2.5 exposure, we measured their effect on the regulation of iNOS and HO-1 expression. PM2.5 increased the nuclear translocation of NF-κB and Nrf2 in BV-2 microglial cells, while ATX treatment inhibited NF-κB and Nrf2 translocation (Figure 6). These results suggest that PM2.5 increases the nuclear translocation of NF-κB and Nrf2, resulting in increased expression of various inflammatory mediators, and that the inhibition of these signals is part of the mechanism underlying the effects of ATX treatment.

### 2.5. Astaxanthin Inhibited Neuronal Cell Death through PM2.5-Induced Microglial Activation

**Figure 7:**
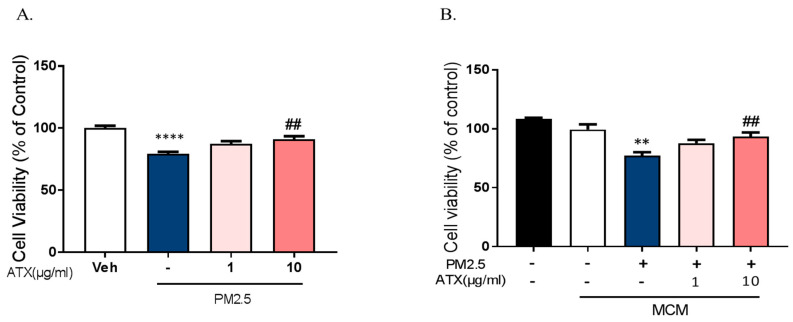
Effects of astaxanthin on PM2.5-induced cell death in rat primary cultured cortical neurons. (**A**) Mixed cultured neurons with glia were treated with ATX (1, 10 µg/mL, astaxanthin) for 4 h. PM2.5 (100 µg/mL) was treated in cultured media for 24 h and was added to MTT solution for MTT assay. **** *p* < 0.0001; veh vs. PM2.5, ## *p* < 0.01; PM2.5 vs. PM2.5 + ATX. (**B**) Rat primary cortical neurons without glia were treated with PM2.5-treated microglial conditioned medium (MCMP) and microglial conditioned media treated with PM2.5 and ATX (MCMA) for 24 h. Data represent means ± SEM. (*n* = 4). ** *p* < 0.01; MCM vs. MCMP, ## *p* < 0.01; MCMP vs. MCMA.

To investigate whether PM2.5 exposure increases neuronal cell death through the microglial activation and whether ATX prevents PM2.5-induced neuronal cell death, we used two model systems: mixed cultured neurons with glia and primary cortical neurons without glia. In mixed cultured neurons with glia, we treated PM2.5 and ATX in culture media. On the other hand, either microglia-conditioned media with PM2.5 (MCMP) or microglial conditioned media with PM2.5 and ATX (MCMA) was added in rat primary cortical neurons. Mixed cultured neuron with glial cells were treated with ATX for 4 h and then added with PM2.5 directly. PM2.5-treated microglial conditioned medium (MCMP) and microglia conditioned medium treated with PM2.5 and ATX (MCMA) were added to rat primary cortical neurons for 24 h. Cell viability was measured by MTT assay. PM2.5 exposure increased neuronal cell death in mixed cultured neurons with glia, while ATX prevented PM2.5-induced toxicity. In addition, MCM stimulated by PM2.5 (MCMP) significantly increased neuronal cell death, while MCM-treated ATX (MCMA) inhibited neuronal cell death (Figure 7). These results indicate that PM2.5 exposure induces neuronal cell death via microglial activation, as well as the fact that ATX treatment can inhibit this PM2.5-induced neuronal damage.

## 3. Discussion

Air pollution is closely associated with the development of several adverse effects on the human body throughout life, having been linked to decrease in function of several organs including the brain, where it has been associated with an increased risk of developing neurodevelopment disorders including autism spectrum disorder and neurodegenerative disorders such as dementia [25,26,27,28]. Recently, studies evaluating the extensive toxicity mediated by air pollution, especially particulate matter (PM2.5), have shown that these particles via several different routes, including a direct way by inhalation or ingestion and indirect routes by systemic responses in the human body, reach the brain and then damage the parenchyma of the CNS through the blood–brain barrier (BBB) [29,30]. In the adult brain, PM2.5 passes through the BBB, inducing the inflammatory responses via (1) glial activation [31,32,33], (2) amyloid toxicity [34,35], and (3) altered expression of the glutamatergic pathway genes [25,36]. Many studies on air pollution have focused on the effects of inhaled pollution in the respiratory systems, especially on the nose and lungs. Interestingly, gestational exposure to air pollutants such as PM2.5 can induce glial activation [33,37], impairment of myelination [38], attenuation of adult neural stem cell [39], and increased observation of depressive behaviors [40]. In this study, we sought to determine whether PM2.5, modeled by DPM, might lead to microglial activation and polarization in the CNS and whether ATX could be a candidate for therapeutic or preventive interventions.

We observed the accumulation of PM2.5 around the microglia in mixed glial cells developed from a combination of rat primary astrocytes and microglia. Microglia are activated in response to various stimuli, including amyloid beta (Aβ), α-synuclein, cytokines, neuronal death, and environmental toxins, especially components of air pollution such as PM2.5 [41,42]. A previous study used diesel exhaust particles (DEP), a type of PM derived from diesel fossil fuels and combustible engines, to show that microglia are activated by PM treatment in vitro [42,43]. Microglia treated with DEP were activated and underwent morphological changes, also displaying an increase of superoxide production, TNFα production, and NO production. Our results indicated that PM2.5 treatment enhanced microglial activation, even after a limited exposure, increasing the mRNA expression of proinflammatory molecular markers, including IL-6, IL-1β, TNFα, iNOS, COX-2, TREM2, and TLR2/4, while decreasing the mRNA expression of anti-inflammatory markers of IL-10 and Arg-1. In this study, our results demonstrated that PM2.5 treatment significantly enhanced the induction of the proinflammatory M1 and DAM phenotype through TREM2 and TLR2/4 and inhibited the development of the anti-inflammatory phenotype. As the first line of defense in the brain, the balance between proinflammatory and anti-inflammatory phenotypes of microglia in the brain is crucial for regulating the inflammatory responses. Previous studies have shown that PM affects pro-inflammatory cytokine release in M1 phenotype and the anti-inflammatory response in M2 phenotype microglia [43,44,45].

Recent studies suggest DAM as a new population of microglia under different disease conditions, which has challenged the classical view of microglia polarization, proinflammatory M1, and immunosuppressive M2 state [12,46,47]. In neurodegenerative diseases such as AD and PD, microglia-mediated neuroinflammation and oxidative stress are involved in detrimental events. Besides the classical category of microglia polarization, recent studies have shown microglia polarization states such as DAM, a recently identified subset of microglia. DAM is a unique phagocytic microglia phenotype associated with disease conditions, including AD, and they have TREM2 (triggering receptor expressed on myeloid cells 2)-dependent roles [48]. Recent findings have shown that TREM2 represents a promising candidate for AD susceptibility and progression, but the association between TREM2 and AD risk was found to be quite different among ethnicities and populations. Endogenous ligands of TREM2 include Aβ, apolipoprotein E, and galectin-3. These ligands induce a microglia polarization to the DAM phenotype. Microglia polarization is increased with oxidative stress in neurodegenerative disease, chronic neuroinflammatory states, and advanced aging. In our study, PM2.5 induced the microglial polarization into proinflammatory M1 state combined with the DAM phenotype. Our results showed that PM2.5 can be a pleiotropic ligand of the DAM phenotype as well as the classical proinflammatory M1 state. Several reports demonstrate that PM2.5 exposure can increase the risk of AD. Our results suggest that PM2.5 exposure can promote the development of neurodegenerative diseases including AD, which is associated with proinflammatory DAM phenotype combined with M1 state and promotes excessive inflammation. In addition, our results prove that ATX can be a potential therapeutic candidate by targeting decreased proinflammatory DAM and increased anti-inflammatory microglia phenotype.

To further investigate the regulatory mediator of M1 polarization induced by PM2.5, we examined the potential roles of iNOS and HO-1, which are known to be inflammatory and anti-inflammatory mediators. Interestingly, our data showed that PM2.5 exposure significantly increased both iNOS and HO-1 expression in BV-2 microglial cells. This indicates that PM2.5 is a potent inducer of inflammation in the CNS. Lipopolysaccharide (LPS), a well-known inflammatory inducer in macrophages as well as microglia, also induces the expressions of iNOS [49] and HO-1 in microglia. Pretreatment with a HO-1 inhibitor, SnPP, significantly increased both LPS-induced iNOS expression and NO production, and inhibition of HO-1 activity by SnPP prevented LPS-induced microglial activation [50]. The interaction between NO and HO-1 plays a critical role in modulating the adaptive response of activated microglia [51], and both iNOS and NO are well known mediators of the inflammatory response in microglial and neuronal cells. Nitric oxide increases oxidative stress by various stimuli and generates more toxic products including peroxynitrite (ONOO^-^), which ultimately leads to neurotoxicity and brain damage. Agents that increase NO production also increase the expression of HO-1, and the production of ONOO^−^ is important in the NO-mediated modulation of HO-1 [51,52].

HO-1 plays a role in the regulation of the inflammation and redox status of cells and its expression is upregulated in response to oxidative stressors such as metal ions and LPS. HO-1 plays a dual role in several cellular responses including oxidative stress, cellular injury, and diseases [50]. HO-1 catabolizes heme into biliverdin/bilirubin, carbon monoxide, and ferrous iron, which commonly play a protective role in various disease conditions including in the development of several neurodegenerative diseases [53,54]. However, recently there has been an increase in the number of studies that describe a more destructive role for HO-1. These studies show that HO-1 is a critical mediator of ferroptosis induction through the excessive accumulation of cellular iron and severe lipid peroxidation, which has been shown to be a causative factor in the progression of several diseases [55,56]. A Janus-faced role for HO-1 has been shown in that both tin(IV)-protoporphyrin (SnPP) as a HO-1 inhibitor and cobaltic(III)-protoporphyrin (CoPP) as a HO-1 activator induce HO-1 expression in rat liver [57]. In this study, PM2.5 (DPM) dramatically increased the expression of inducible HO-1 and iNOS/NO. Treatment with HO-1 inhibitor SnPP inhibited the PM2.5-induced cell death (data not shown), and our data indicate that increased HO-1 expression by PM2.5 augments inflammation and reinforces the feedback loops, trapping the cells in hyperinflammation through a vicious cycle.

Further, our results indicate that PM2.5 induced HO-1 protein expression via the Nrf2 transcription factor and that ATX treatment decreased both Nrf2 and HO-1 expression. NF-E2-related factor 2 (Nrf2) is a transcription factor that plays a critical role in defending against oxidative stress by increasing the production of antioxidant enzymes such as heme oxygenase-1 (HO-1) and NAD(P)H: quinone oxidoreductase 1 (NQO1) [17,58]. Recently, it has been reported that Nrf2 has a pivotal role in inflammation [59]. Nrf2 is involved in inflammatory diseases via regulating NLR family pyrin domain containing 3 (NLRP3) inflammasome activity. Additionally, the activation of the Nrf2 pathway is associated with pro-inflammatory signaling pathways and the inhibition of the NF-κB signaling pathway [60]. Decreases of Nrf2 after exposure to DEP extracts (DEPe) maintained the LPS-induced IL-6 expression in macrophages [45]. ATX decreased LPS-induced Nrf2 mRNA expression, however, Nrf2 nuclear translocation was increased following ATX treatment in LPS-stimulated Raw264.7 macrophages [17,24,61]. These different cellular functions may be related to differences in the various regulatory pathways in these cell models. In a mouse sepsis model, Nrf2 disruption after DEPe exposure was related to changes in the regulation of IL-6 expression through NF-κB activation [62]. In this study, we found that ATX repressed PM2.5-stimulated Nrf2 protein expression in BV-2 microglial cells, and a previous study showed that H_2_O_2_ treatment induced endogenous Nrf2 expression in rat cardiomyocytes [63]. Many reports have demonstrated that Nrf2 plays a crucial role in regulating intracellular redox homeostasis by activating the antioxidative system [17,64]. Macrophages from Nrf2 Knockout (KO) mice showed no increase in ROS production following LPS stimulation. However, LPS treatment increased the inflammatory response via increased IL-6 and IL-1β mRNA expression in Nrf2 KO mice. These results demonstrate that Nrf2 also plays a critical role in mediating the inflammatory response, although it can prevent ROS accumulation and oxidative stress by activating the antioxidant enzymes. In this study, our results show that ATX treatment decreased the inflammatory response induced by PM2.5 treatment in BV-2 microglia. Surprisingly, the inhibitory effects of ATX treatment on PM2.5-induced inflammatory responses might be mediated by the inhibition of inflammatory mediators such as HO-1 and iNOS via changes in the expression of Nrf2 and NF-κB. Therefore, our results indicate that Nrf2 might be a critical player in the mediation of the inflammatory response, despite its potential role in the antioxidative response facilitated by HO-1.

## 4. Materials and Methods

### 4.1. Materials

Materials used in this study were sourced as follows: Dulbecco’s modified Eagle’s medium (DMEM)/F12, neurobasal medium (NBM), penicillin–streptomycin (P/S), 0.25% trypsin- ethylenediaminetetraacetic acid (EDTA), horse serum (HS), and fetal bovine serum (FBS) were sourced from Gibco BRL (Grand Island, NY, USA). Dimethyl sulfoxide (DMSO) was sourced from Invitrogen (Carlsbad, CA, USA), and ECL Western blotting detection reagent was bought from iNtRON Biotech (Seoul, Korea). The PM2.5 (diesel particulate matter) (NIST1605b), lipopolysaccharide (LPS; O111:B4), and anti-β-actin were sourced from Sigma-Aldrich (St. Louis, MO, USA), while the anti-iNOS was sourced from Abcam (Cambridgeshire, United Kingdom). The anti-phospho-ERK, anti-phospho-JNK, anti-phospho-p38, anti-phospho-Akt, anti-ERK, anti-JNK, anti-p38, and anti-Akt antibodies were all sourced from Cell Signaling Technology (Danvers, MA, USA), and the anti-heme oxygenase-1 (HO-1) was bought from Invitrogen.

### 4.2. Cell Culture and Drug Treatment

BV-2 cell lines were derived from immortalized murine neonatal microglia and the cells were grown in high glucose Dulbecco’s modified Eagle’s medium (DMEM) supplemented with 5% heat-inactivated FBS, 100 units/mL penicillin, 100 mg/mL streptomycin, and 2 mM glutamine (Gibco BRL, Grand Island, NY, USA). The cells were seeded at 2 × 10^5^ cells/mL in 24-well or 6-well plates (TPP, Switzerland) and incubated at 37 °C in humidified 5% CO_2_ and 95% air.

For mixed cortical neuron culture with glia, we purchased pregnant Sprague-Dawley rats from Orient Bio (Gyeonggi, Korea), and primary cortical neurons were isolated from embryonic day-17 (E17) cortices of Sprague Dawley (SD) rat embryos, as described previously [65]. Briefly, the cortex was mechanically dissociated and gently triturated in the culture medium (minimal essential media). Then, the cells were seeded at 2 × 10^7^ cells/mL onto 50 µg/mL poly-d-lysine (PDL)-coated plates in the culture medium supplemented with 5% HS, 5% FBS, and 2 mM L-glutamine. The cultures were maintained at 37 °C in a humidified 5% CO_2_ incubator and cultured media was changed every 2 days. The cultured cells were used after 8 days of in vitro differentiation.

Primary cortical neurons were isolated from the cerebral cortex of embryonic day 17 (E17) SD rats. Cortical cell suspension was seeded on the poly-D-lysine-coated plate (50 μg/mL) and incubated at 7 × 10^5^ cells/mL onto 50 µg/mL poly-d-lysine (PDL)-coated plates in NBM with B27 and L-glutamine in a 95% CO_2_ incubator at 37 °C for 10 days, and media were half-replaced with fresh media every 3 days.

Mixed rat glial cultures were prepared from the prefrontal cortices of 2-day-old AD rat pus. Cell suspensions were seeded onto poly-D-lysine (20 μg/mL)-coated plates and the cultures were maintained in DMEM/F12 with 10% heat-inactivated FBS, 100 U/mL penicillin, and 100 mg/mL streptomycin. Confluent cells were sub-cultured by re-plating at low density (2.5 × 10^5^ cells/mL) in 24-well or 6-well plates (TPP, Trasadingen, Switzerland). Cells that reached confluence at 5 days after subculture were used for this study. Animal experimental procedures were carried out following the protocols approved by the Institutional Animal Care and Use Committee (IACUC) of Konkuk University (KU19137).

Astaxanthin (ATX) was provided as 97% *Haematococcus pluvialis* extracts from Korean Drug Co., LTD (Seoul, Korea). Cultured cells were treated with 1–10 μg/mL of ATX or vehicle for 4 h and then added with 100 μg/mL of PM2.5 (diesel particulate matter, Sigma-Aldrich, St. Louis, MO, USA) for the indicated time with or without ATX. ATX was handled in the dark to prevent any light-stimulated degradation and was freshly diluted with DMSO for each experiment.

### 4.3. Determination of Nitrite Concentration

NO production was determined by measuring nitrite production, as described previously [66,67]. Briefly, Griess reagent was freshly prepared by mixing equal volumes of 0.1% napthylethylenediamine dihydrochlroride and 1% sulfanilamide in 5% phosphoric acid and then added to spent culture media for 5 min. These mixtures were then evaluated for absorbance at 540 nm using a UV spectrophotometer (Spectramax 190, Molecular device, Palo Alto, CA, USA), and NO was quantified by using a sodium nitrite (1–80 μM) standard curve [68].

### 4.4. Measurement of Intracellular ROS

Intracellular ROS formation was measured by fluorescence detection using 2′,7′-dichlorodihydrofluorescein diacetate (H2DCF-DA; Invitrogen, Carlsbad, CA, USA) [69]. This non-fluorescent dye freely permeates into cells, where it de-esterifies to form the ionized-free acid (DCFH), which reacts with ROS to form fluorescent 2′,7′-dichlorofluorescein (DCF). After the PM2.5 treatment, we washed the cells with Phosphate Buffered Saline (PBS), loaded 20 µM of H2DCF-DA for 30 min at 37 °C, and then washed them again with PBS. DCF fluorescence was analyzed using a fluorescence plate reader (Gemini EM, Molecular Devices, Palo Alto, CA, USA) at an excitation of 490 nm and an emission of 530 nm.

### 4.5. Quantitative Reverse Transcription Polymerase Chain Reaction (qRT-PCR)

Expression of *Il*-1*β*, *Il*-6, *TNFα*, *IL*-10*, Arginase*-1*, iNOS*, *COX*-2*, Trem*2*, Tlr*2 and *Tlr*4*, HO*-1, and *Gapdh* in treated BV-2 microglial cells was determined by qRT-PCR. RNA was extracted using Trizol reagent (Invitrogen, Carlsbad, CA, USA) and concentration was measured using a spectrophotometer (Nanodrop Technologies, Wilmington, NC, USA). cDNA synthesis was performed using 0.5 µg of total RNA and an RT reaction mixture including RevertAid Reverse transcriptase, reaction buffer (Thermo Fisher Scientific, Waltham, MA, USA), and dNTP (Promega, Madison, WI, USA). The gene-specific primer pairs used in this analysis are described in Table 1.

qRT-PCR was carried out using a 20 µL reaction system containing 5 µL cDNA, 1.2 µL of each primer set at a final concentration of 10 pM, 3.8 µL distilled water (DW), and 10 µL BrightGreen 2x qPCR Master Mix (Applied Biological Materials Inc., Richmond, Canada) on a QuantStudio3 Real-Time PCR System (A28132, Applied Biosystems, Foster City, CA, USA) using the following parameters: [94 °C, 30 s; 60 °C, 1 min; 72 °C, 30 s] × 30 cycles then 72 °C for 10 min for *Il*-1*β*, *Il*-6, *TNFα*, *iNOS, COX*-2*, Trem*2*, Tlr*2, and *Tlr*4, and [94 °C, 30 s; 60 °C, 1 min; 72 °C, 30 s] × 23 cycles then 72 °C, 10 min for *Gapdh*. All assays including those for the controls were performed in triplicate. The expression levels of GAPDH and 18 s rRNA were used as internal controls and the relative expression of each transcript was calculated using the 2^-^ΔΔCT formula to the fold change as described the ABI user guide.

### 4.6. Western Blot Analysis

After we treated cells with PM2.5 and ATX, they were harvested in radioimmunoprecipitation assay [70] buffer consisting of 2 mM EDTA, 0.1% (w/v) SDS, 50 mM Tris-HCl, 150 mM sodium chloride, 1% Triton X-100, and 1% (w/v) sodium deoxycholate. Total proteins was quantified using a Bicinchoninic acid (BCA) assay kit (Thermo Fisher Scientific, MA, USA) and boiled for 5 min at 100 °C. A total of 10 µg of protein from each sample was loaded onto 10% SDS-polyacrylamide gel and then subjected to electrophoresis (SDS-PAGE) at 100 V for 120 min. Separated proteins were then transferred to the nitrocellulose membranes for 90 min and the blots were blocked with 5% skim milk in Tris-buffered saline with 0.1% Tween 20 (TBST) for 1 h at room temperature. Blots were then washed with Tris-buffered saline and 0.1% Tween 20 (TBS-T) and incubated in their appropriate primary antibody, β-actin (1:40,000), iNOS (1:2000), HO-1 (1:10,000), p-pERK/ERK/p-p38/p38/p-JNK/JNK (1:1000), and NF-κB p65 (1:1000) overnight at 4 °C. Then, blots were washed three times and incubated with horseradish peroxidase (HRP)-conjugated secondary antibodies (Life Technologies, Carlsbad, CA, USA) at room temperature for 60 min. The bands were detected using the enhanced chemiluminescence detection system (iNtRON Biotech., Seoul, Korea) and visualized on a LAS-3000 image detection system (Fuji, Japan). The bands were quantitated with ImageJ system and β-actin was used as the loading control.

### 4.7. Measurement of Cell Viability

BV-2 microglial cells were cultured in 24-well plates with each treatment group represented by 4 wells. After we treated them with different concentrations of PM2.5 (5, 25, 50, 100 µg/mL), cells were incubated for a further 24 h and then evaluated for cell viability using the 3-[4,5-dimethylthiazol-2-yl]-2,5-diphenyl-tetrazolium bromide (MTT) assay. MTT is a water-soluble tetrazolium salt that is reduced by metabolically viable cells to a colored, water-insoluble formazan salt. MTT (5 mg/mL) was added to the cell culture medium and then incubated at 37 °C for 2 h in a 5% CO_2_ atmosphere. Then, the MTT-containing medium was replaced with DMSO and the absorbance was read at 570 nm with a microplate reader (Spectramax 190, Molecular Devices, Palo Alto, CA, USA). The percentage of surviving cells was calculated compared to the control (untreated cells) [71].

### 4.8. Immunocytochemistry

To investigate the microglial activation, we washed cultured BV-2 cells twice with PBS and fixed them with 4% paraformaldehyde for 20 min. Samples were then permeabilized in permeabilization buffer (0.3% Triton X-100 in PBS) at room temperature for 15 min and then blocked using blocking buffer (1% BSA, 5% FBS in PBS) for 1 h at room temperature. Samples were then incubated with their appropriate primary antibody against microglia (Iba-1, 1:500; FUJIFILM Wako Pure Chemical Corp. Osaka, Japan) overnight at 4 °C and rinsed with washing buffer (1.5% HS, 0.1% Triton X-100 in PBS) 3 times for 10 min. Samples were then treated with secondary antibodies conjugated with Alexa488 (Invitrogen, Carlsbad, CA, USA) in blocking buffer and incubated for 2 h at room temperature. After we washed them 3 times with PBS, samples were incubated with ToPro3 (Invitrogen, Carlsbad, CA, USA) to allow for nuclear staining, mounted using Vectashield (Vector laboratories, Burlingame, CA, USA) and observed using a confocal microscope (LSM900, Carl Zeiss, Oberkochen, Germany). Images were captured at 3 random areas per well and 3 wells per group. To measure the change of microglial morphology, we counted the number of activated microglia in captured picture of phase contrast using ImageJ system. Activated microglia is characterized by over 20 μm length of cytoplasmic processes with maintained high intensity of Iba-1 expression.

### 4.9. Statistical Analysis

Experimental results are expressed as the mean ± SEM. Statistical comparisons were performed using one-way ANOVA and *t*-test using GraphPad Prism 5 software (San Diego, CA, USA), and a value of *p* < 0.05 was considered significant.

## 5. Conclusions

In a summary, the present study demonstrated that PM2.5 induces the proinflammatory M1 and DAM phenotype and suppresses the anti-inflammatory phenotype by upregulating iNOS and HO-1 expression in BV-2 microglia. NF-κB and Nrf2 signaling may be involved in neuroinflammation via their roles in proinflammatory M1 and DAM polarization. In addition, ATX treatment inhibited the mRNA expression of inflammatory cytokines and upregulated anti-inflammatory factors. These findings suggest that ATX could be a potential candidate for preventing PM2.5-induced neuroinflammation. A better understanding of the regulatory and signaling relationships in neuroinflammation and the discovery of other ATX-like phytochemicals may provide a therapeutic strategy for attenuating the effects of PM2.5-induced brain damage.

## Figures and Tables

**Table 1:** Gene-specific primers used in this study.

	Sense	Antisense
*Il*-1*β*	AAA ATG CCT CGT GCT GTC TG	CTA TGT CCC GAC CAT TGC TG
*Il*-6	TTG TGC AATGGC AAT TCT GA	TGG AAG TTG GGG TAG GAA GG
*TNFα*	TAG CCC ACG TCG TAG CAA AC	GGA GGC TGA CTT TCT CCT GG
*iNOS*	CTG GCT GCC TTG TTC AGC TA	AGT GTA GCG TTT CGG GAT CT
*COX*-2	TGCTGTACAAGCAGTGGCAA	AGGTGCTCGGCTTCCAGTAT
*HO*-1	TGTCACCCTGTGCTTGACCT	ATACCCGCTACCTGGGTGAC
*IL*-10	AGGCGCTGTCATCGATTTCT	ATGGCCTTGTAGACACCTTGG
*Arg*-1	ACAAGACAGGGCTCCTTTCAG	CGTTGAGTTCCGAAGCAAGC
*Trem*2	GACCTCTCCACCAGTTTCTCC	TACATGACACCCTCAAGGACTG
*Tlr*2	TGCTTTCCTGCTGGAGATTT	TGTAACGCAACAGCTTCAGG
*Tlr*4	ACCTGGCTGGTTTACACGTC	CTGCCAGAGACATTGCAGAA
18*S rRNA*	CATTAAATCAGTTATGTTGGTTCCTTTGD	TCGGCATGTATTAGCTCTAGAATTACC
*Gapdh*	GTG AAG GTC GGT GTG AAC GGA TTT	CAC AGT CTT CTG AGT GGC AGT GAT
